# Identification of Tumor Microenvironment-Related Prognostic lncRNAs in Lung Adenocarcinoma

**DOI:** 10.3389/fonc.2021.719812

**Published:** 2021-08-02

**Authors:** Ligong Yuan, Feng Li, Shuaibo Wang, Hang Yi, Fang Li, Yousheng Mao

**Affiliations:** Department of Thoracic Surgery, National Cancer Center/National Clinical Research Center for Cancer/Cancer Hospital, Chinese Academy of Medical Sciences & Peking Union Medical College, Beijing, China

**Keywords:** lung adenocarcinoma, lncRNAs, LASSO regression, immune microenvironment, prognostic biomarkers

## Abstract

**Background:**

Lung adenocarcinoma (LUAD) is the most common type of lung cancer and is a severe threat to human health. Although many therapies have been applied to LUAD, the long-term survival rate of patients remains unsatisfactory. We aim to find reliable immune microenvironment-related lncRNA biomarkers to improve LUAD prognosis.

**Methods:**

ESTIMATE analysis was performed to evaluate the degree of immune infiltration of each patient in TAGA LUAD cohort. Correlation analysis was used to identify the immune microenvironment-related lncRNAs. Univariate cox regression analysis, LASSO analysis, and Kaplan Meier analysis were used to construct and validate the prognostic model based on microenvironment-related lncRNAs.

**Results:**

We obtained 1,178 immune microenvironment-related lncRNAs after correlation analysis. One hundred and eighty of them are independent prognostic lncRNAs. Sixteen key lncRNAs were selected by LASSO method. This lncRNA-based model successfully predicted patients’ prognosis in validation cohort, and the risk score was related to pathological stage. Besides, we also found that TP53 had the highest frequency mutation in LUAD, and the mutation of TP53 in the high-risk group, which was identified by our survival model, has a poor prognosis. lncRNA-mRNA co-expression network further suggested that these lncRNAs play a vital role in the prognosis of LUAD.

**Conclusion:**

Here, we filtered 16 key lncRNAs, which could predict the survival of LUAD and may be potential biomarkers and therapeutic targets.

## Introduction

The incidence of lung cancer exceeds 2 million each year, of which approximately 1.8 million ultimately die, making it the leading cause of cancer-related deaths worldwide (1). Of these, 85% of lung cancers are diagnosed as non-small cell lung cancer (NSCLC), and 60% of the patients have locoregional advance or distant metastases (2). There are two major subgroups of NSCLC, LUAD and LUSC. LUAD and LUSC are distinct at the transcriptome level and in terms of cellular control networks (3, 4). In addition, LUAD shows different genetic drivers and different prognostic profiles compared with LUSC (4, 5). Numerous therapeutic clinical trials in NSCLC have shown that LUAD patients showed different responses compared with LUSC (3, 6). This suggests that LUAD and LUSC are different at pathological and molecular levels. Therefore, the development of new and more effective subtype-specific molecules and associated targeted therapies is of great significance for NSCLC. LUAD is the most common type of lung cancer in nonsmokers, although it can occur in smokers. LUAD morphologic types include glandular alveolar, papillary, solid, micropapillary, and invasive mucinous types (7). In addition, it is more common in women than in men and is more likely to occur in younger people and to present in a more advanced stage (8). LUAD has been the most common histological subtype of lung cancer in the last few decades (9). Lung adenocarcinoma (LUAD) is a severe threat to human health, with more than 1 million deaths per year worldwide (1, 10). Although many therapies have been applied to LUAD, the long-term survival rate of patients remains unsatisfactory, with an average 5-year survival rate of 16% (11, 12).

Recently, there is a growing body of opinion that the immune cell plays an essential role in tumor (13). Cancer is often able to evade different components of the immune system, and the immune microenvironment is a critical factor associated with cancer progression (14). Moreover, many studies have shown that immune-related parameters can predict the prognosis of LUAD patients (15, 16). Therefore, we need a reliable immune microenvironment-related biomarkers to assess LUAD prognosis to guide in the therapeutic management.

LncRNAs are transcripts with non-coding potential and have more than 200 nucleotides (17). The current understanding of the function of lncRNA remains largely unclear. LncRNAs may regulate the expression level of genes by post-transcriptional regulation (18). Meanwhile, lncRNAs may further influence tumor cell migration by regulated target genes (17). However, the immune-related lncRNA signature of lung cancer is still not widely used.

Here, we obtained immune microenvironment-related lncRNAs and evaluated the prognostic efficacy through a mass of bioinformatic analysis. We obtained several lncRNAs that could predict LUAD prognosis, and we also established a lncRNA-mRNA co-expression network to investigate the mechanism of these lncRNAs in LUAD.

## Materials and Methods

### Gene Expression Data Sets for Lung Cancer

Data from two publicly available data sets were incorporated into our study. The gene expression data, genomic mutation data, and corresponding clinical information of samples from patients with lung adenocarcinoma (LUAD) were downloaded from the TCGA database. We randomly extracted half of the LUAD samples as training cohort, and the rest of LUAD samples as validation cohort (**Table S1**).

Gene expression microarray of lung cancer (GSE30219, GSE37745, and GSE31210) with corresponding overall survival (OS) data was downloaded from GEO and served as the validation data set. Gene expression data of all three data sets were normalized by Robust Multichip Average (RMA) method using “affy” package in R.

### Identification of Immune-Related lncRNAs

We evaluated tumor immune infiltration of TCGA LUAD training cohort based on ESTIMATE (Estimation of STromal and Immune cells in MAlignant Tumor tissues using Expression data) method by R software with package “estimate” (19). Next, we used Pearson correlation analysis to identify immune-related lncRNAs based on ESTIMATE scores in TCGA LUAD training cohort. A total of 1178 immune-related lncRNAs with P < 0.001 and |cor| > 0.2 were finally identified.

### Identification of Prognosis-Related lncRNAs and Prognosis Model

A univariate cox regression analysis was performed to select OS-related lncRNAs from abovementioned 1178 lncRNAs in the TCGA LUAD training cohort. A total of 180 immune- and prognosis-related lncRNAs were screened out with *P* < 0.05. Next, we performed the LASSO analysis to select the crucial variables and to filter potential survival model, and a model consisting of 16 lncRNAs was identified. Ultimately, the risk score of each patient based on 16 lncRNA expression was calculated by the following formula: risk score = (exp _lncRNA1_ × CC_lncRNA1_) + (exp _lncRNA2_ × CC_lncRNA2_) + … + (exp _lncRNA16_ × CC_lncRNA16_). CC is the coefficient calculated by LASSO. Patients were divided into the high- and low-risk groups based on the median value.

### Pathological, Immune, and Genomic Association Analysis for Risk Group

Wilcoxon rank-sum test was performed to calculate the risk scores’ difference between clinical pathological stage groups (TNM Classification of Malignant Tumors). The differences in ESTIMATE scores between high- and low-risk groups in LUAD training cohort were calculated by the same method. Then, we proceed the expression analysis of five immune checkpoint, including PDCD1 (code PD-1), CD274 (code PD-L1), CTLA-4, CD47, and BTLA. Wilcoxon rank-sum test was also used for exploring the risk difference of the five immune checkpoint in TCGA LUAD training cohort. We also analyzed genomic mutation status between high-risk group and low-risk group in TCGA LUAD training cohort and sorted genes according to mutation frequency.

### Establish lncRNA-mRNA Co-Expression Network and Functional Analysis

Pearson correlation analysis was performed for constructing the co-expression network of the 16-lncRNAs, the co-expression relationships with P < 0.001, cor > 0.3 were retained. We calculated the degree in the network for each lncRNA. We visualized the significant co-expression relationships and retained the mRNAs, which have significant co-expression relationships with more than four lncRNAs. A total of 225 mRNAs were retained for subsequent functional analysis.

We performed pathway and process enrichment analysis for 225 mRNAs with many ontology sources, such as KEGG pathway, GO biological processes, reactome gene sets, and canonical pathways through Metascape web-based tool. Parameters are selected as P < 0.01, the terms’ minimum count is set at three, and the enrichment factor > 1.5.

### Survival Analysis

Kaplan-Meier analysis was performed to calculate the difference in the survival time between high-risk and low-risk patients. Survival analysis was used to assess the difference in the survival time between the two groups. P < 0.05 was regarded as statistically significant.

## Result

### Identification of lncRNAs Associated With Immune Infiltration

**Figure 1:**
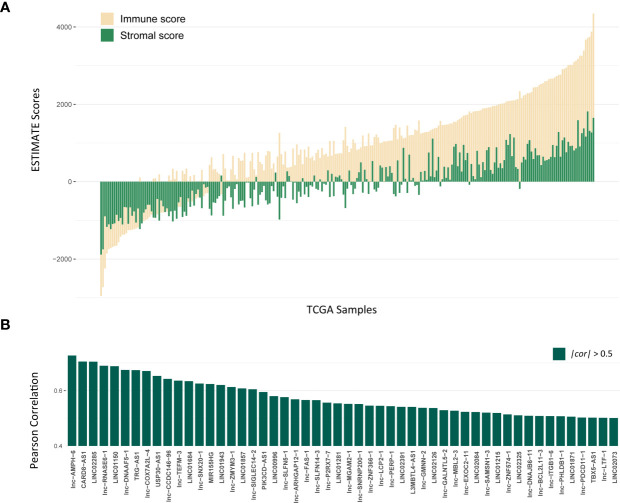
Identification of lncRNAs associated with Immune infiltration. **(A)** The distribution of ESTIMATE scores of TCGA LUAD training cohort, yellow bars mean immune scores and green bars mean stromal scores. **(B)** The correlation for 52 lncRNAs with P < 0.001 and |cor| > 0.5 by Pearson correlation analysis.

The workflow of this study is shown in **Figure S1**. To explore lncRNAs, which function in tumor immune infiltration, we first used the ESTIMATE method to assess the level of immune cell infiltration in the TCGA LUAD training cohort (**Figure 1A**). We analyzed the correlation between ESTIMATE scores and the expression of lncRNAs and identified 1178 lncRNAs significantly associated with ESTIMATE scores. **Figure 1B** showed most significantly associated 52 lncRNAs with P < 0.001 and |cor| > 0.5 by Pearson correlation analysis.

### Identification of Prognosis-Related lncRNAs and Construction of 16-lncRNAs Prognostic Model

**Figure 2:**
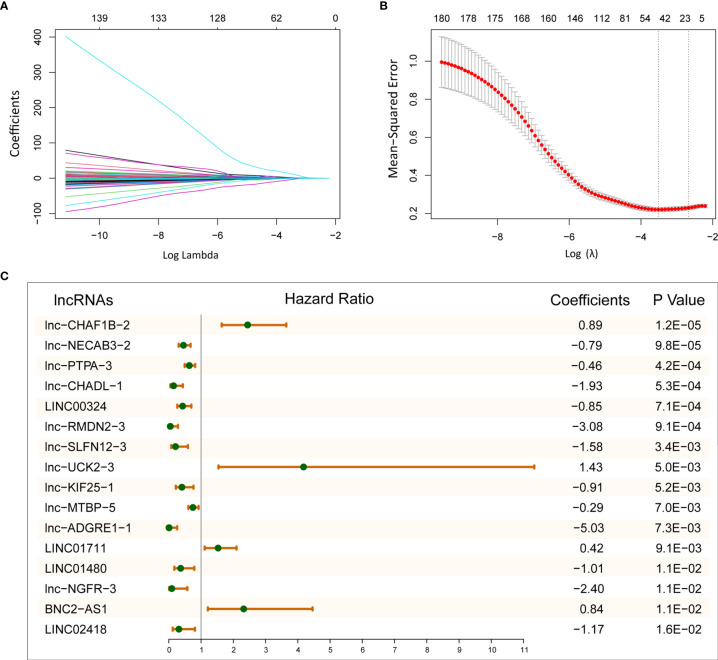
Establish 16-lncRNAs prognostic model. **(A)** LASSO coefficient profiles of 180 immune- and prognosis-related lncRNAs. **(B)** Cross-validation for tuning parameter selection in the LASSO model. **(C)** After LASSO selection, 16 lncRNAs were chose for constructing prognostic model.

To explore the prognostic efficacy of selected immune-related lncRNAs, we performed univariate Cox proportional regression analysis and obtained 180 immune-related lncRNAs, which were significantly related to OS. Next, LASSO analysis was used to filter the potential survival model. A model with 16 immune- and prognosis-related lncRNAs was constructed (lnc-CHAF1B-2, lnc-NECAB3-2, lnc-PTPA-3, lnc-CHADL-1, LINC00324, lnc-RMDN2-3, lnc-SLFN12-3, lnc-UCK2-3, lnc-KIF25-1, lnc-MTBP-5, lnc-ADGRE1-1, LINC01711, LINC01480, lnc-NGFR-3, BNC2-AS1, and LINC02418) (**Figures 2A, B**). Then, based on the TCGA LUAD training set, we established a predictive model: risk score = (0.89 × lnc-CHAF1B-2 exp) + (−0.79 × lnc-NECAB3-2 exp) + (−0.46 × lnc-PTPA-3 exp) + (−1.93 × lnc-CHADL-1 exp) + (−0.85 × LINC00324 exp) + (−3.08 × lnc-RMDN2-3 exp) + (−1.58 × lnc-SLFN12-3 exp) + (1.43 × lnc-UCK2-3 exp) + (−0.91 × lnc-KIF25-1 exp) + (−0.29 × lnc-MTBP-5 exp) + (−5.03 × lnc-ADGRE1-1 exp) + (0.42 × LINC01711 exp) + (−1.01 × LINC01480 exp) + (−2.40 × lnc-NGFR-3 exp) + (0.84 × BNC2-AS1 exp) + (−1.17 × LINC02418 exp) (**Figure 2C**).

### The Relationship Between Risk Scores With Clinical Pathological Features

**Figure 3:**
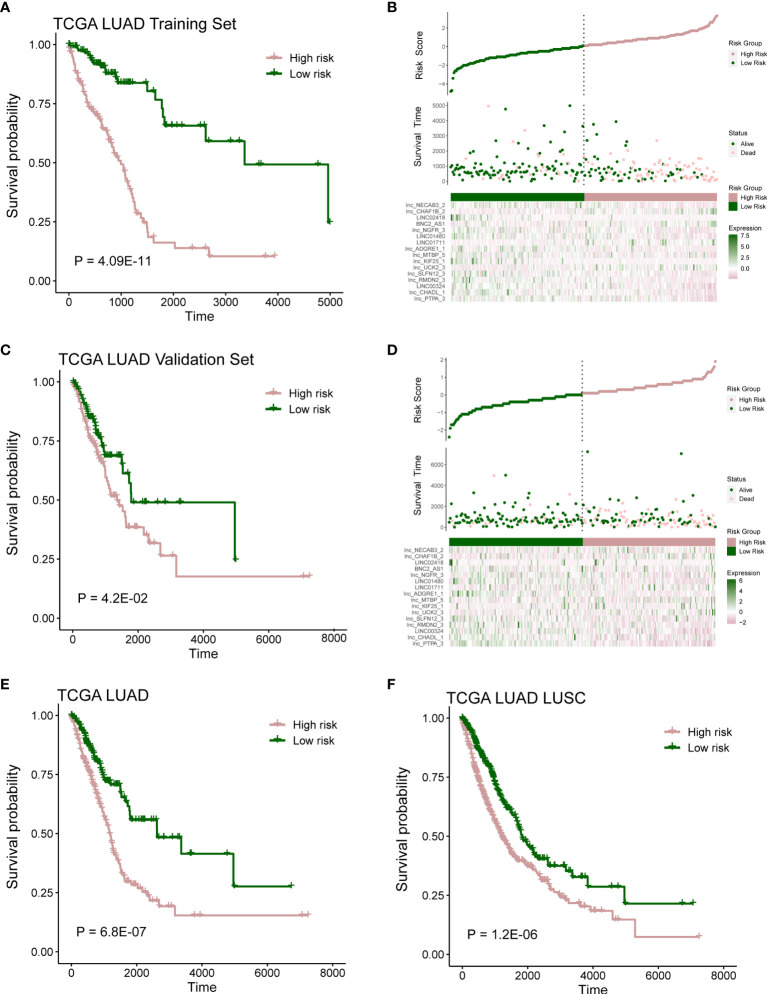
Prognostic model capable of distinguish patients. **(A)** Survival analysis between high- and low-risk samples in TCGA LUAD training cohort. **(B)** Distribution of survival time and risk scores, which were calculated based on the expression of 16-lncRNA prognostic model in TCGA LUAD training cohort. **(C)** Survival analysis in TCGA LUAD validation cohort. **(D)** Distribution of survival time and risk scores, which were calculated based on the expression of 16-lncRNA prognostic model in TCGA LUAD validation cohort. **(E, F)** Survival analysis in TCGA LUAD cohort or TCGA lung cancer (including LUAD and LUSC) cohort.

According to the risk score of LASSO analysis, we distinguished the patients into low-risk and high-risk groups. In training cohort, patients in the high-risk group had a poor OS (**Figure 3A**; P = 4.09E-11; log-rank test), and there are more alive patients in the low-risk group (**Figure 3B**). In validation cohort, the high-risk group also had a poor prognosis (**Figure 3C**; P = 4.2E-02; log-rank test), and the alive patients in the high-risk group were less than the low-risk group (**Figure 3D**). The same observation was also found in the whole TCGA LUAD (training set and validation set) and TCGA lung cancer (LUAD and LUSC) cohorts (**Figures 3E, F** and **Figures S2A, B**; P = 6.8E-07, P = 1.2E-02; log-rank test).

**Figure 4:**
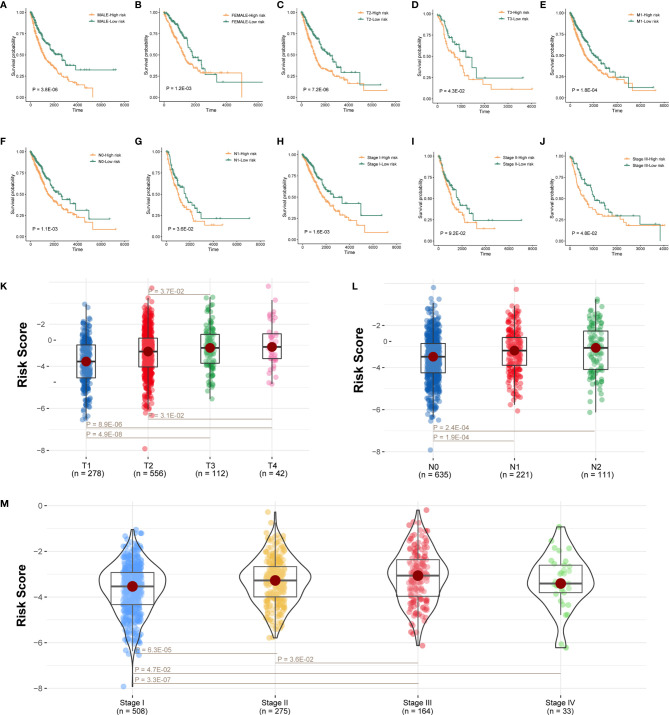
Prognostic model connected with clinical pathological features. **(A–J)** Survival analysis of sexual groups, T stage (T2 and T3) groups, M3 stage group, N stage (N0 and N1) groups, or clinical stage (stage I, stage II, and stage III) groups in TCGA lung cancer cohort. **(K**–**M)** Wilcoxon rank-sum test showed the risk differences between T stage groups, M stage groups, or clinical stage in TCGA lung cancer cohort.

Next, further investigation was conducted to determine whether the risk scores could indicate prognosis in different subgroups of clinical features. In the gender subgroup, high-risk patients had a poor prognosis (**Figures 4A, B**; P < 0.05; log-rank test). Similarly, in the T subgroups (T2 and T3), M1 subgroup, N subgroups (N0 and N1), and pathological stage (stage I and stage III), high-risk score patients had a significantly poor survival (**Figures 4C–H, J**; P < 0.05; log-rank test), and stage II subgroup has the same trend but without statistical significance (**Figure 4I**; P = 9.2E-02; log-rank test).

We also analyzed the difference of risk score among tumor grade groups. The risk scores in stage T2 and T3 were significantly higher than those in stage T1, and the risk scores in stage T3 and T4 were higher than stage T2 (**Figure 4K**; P < 0.05). Besides, the risk scores in stage N1 and N2 were higher than stage N0 (**Figure 4L**; P < 0.05). Moreover, the risk scores in stage II, stage III, and stage IV were significantly higher than stage I, and the risk scores in stage III were also higher than stage II (**Figure 4M**; P < 0.05).

### Immune Infiltration and Genomic Mutation Discrepancy in Different Risk Groups

**Figure 5:**
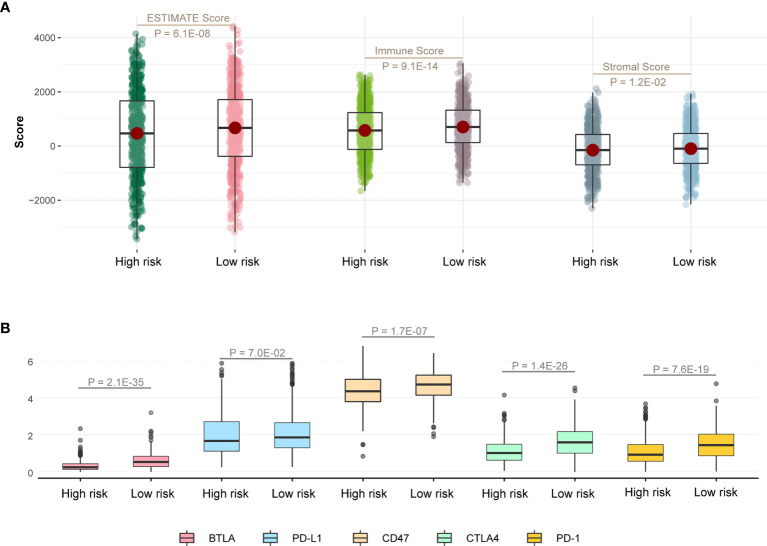
Immune infiltration discrepancy in different risk groups. **(A)** One-sided Wilcoxon rank-sum test showed the differences of ESTIMATE scores between high- and low-risk groups in TCGA LUAD training cohort. **(B)** The differences in the expression of five immune checkpoint related genes between high- and low-risk groups in TCGA LUAD training cohort.

At first, we calculated ESTIMATE scores (immune scores and stromal scores) in high-risk and low-risk groups. We found that the high-risk score group had lower immune scores, stromal scores, and ESTIMATE scores compared with the low-risk score group (**Figure 5A**; P < 0.05). Next, we evaluated the expression difference of five immune checkpoint between high-risk score group and low-risk score group. The expressions of BTLA (P = 2.1E-35), CD47 (P = 1.7E-07), CTLA4 (P = 1.4E-26), and PD-1 (P = 7.6E-19) in low-risk group were significantly greater than those in the high-risk group (**Figure 5B**), and expression of PD-L1 has the same trend but no statistical significance (**Figure 5B**; P = 7.0E-02).

**Figure 6:**
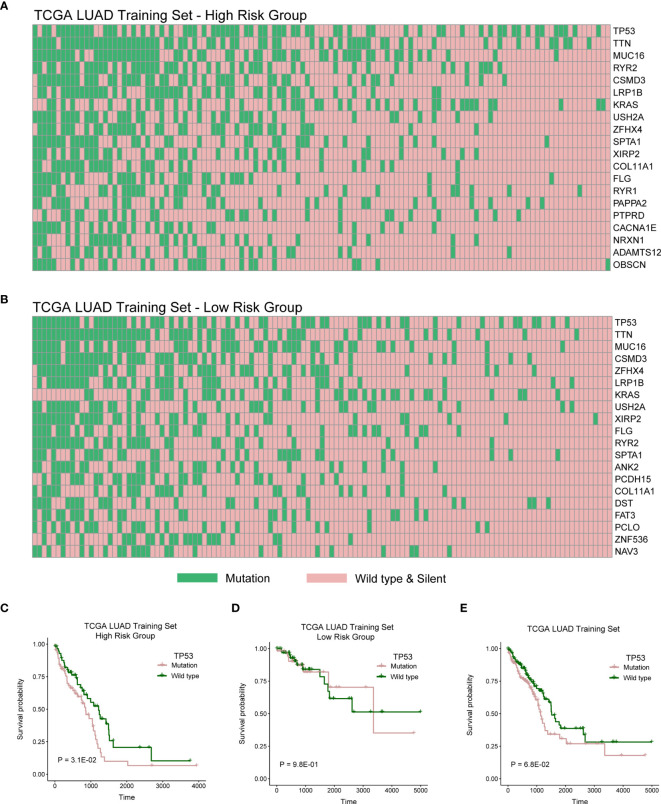
High- and low-risk groups with different distribution of mutation. **(A)** The distribution of top 20 genes with high frequency in high-risk group of TCGA LUAD training cohort. **(B)** The distribution of top 20 genes with high frequency in low-risk group of TCGA LUAD training cohort. **(C–E)** Log-rank test was used to assess the difference in OS between TP53 mutation and wild type samples in TCGA LUAD training cohort with high risk, TCGA LUAD training cohort with low risk, or TCGA LUAD training cohort.

We then explored genomic mutation status between high- and low-risk groups. **Figures 6A, B** showed the top 20 mutations in TCGA cohort. TP53 is the most frequent mutation gene in both groups. We next investigated relationship between the mutation of TP53 and OS in the high- and low-risk groups and found the mutation of TP53 indicated poor prognosis in the high-risk group (**Figure 6C**; P = 3.1E-02), but there was no distinction in the low-risk group (**Figure 6D**; P = 9.8E-01), and there has a trend but no statistical significance in whole TCGA LUAD cohort (**Figure 6E**; P = 6.3E-02).

### Hub lncRNAs in Co-Expression Network Were Associated With Prognosis

**Figure 7:**
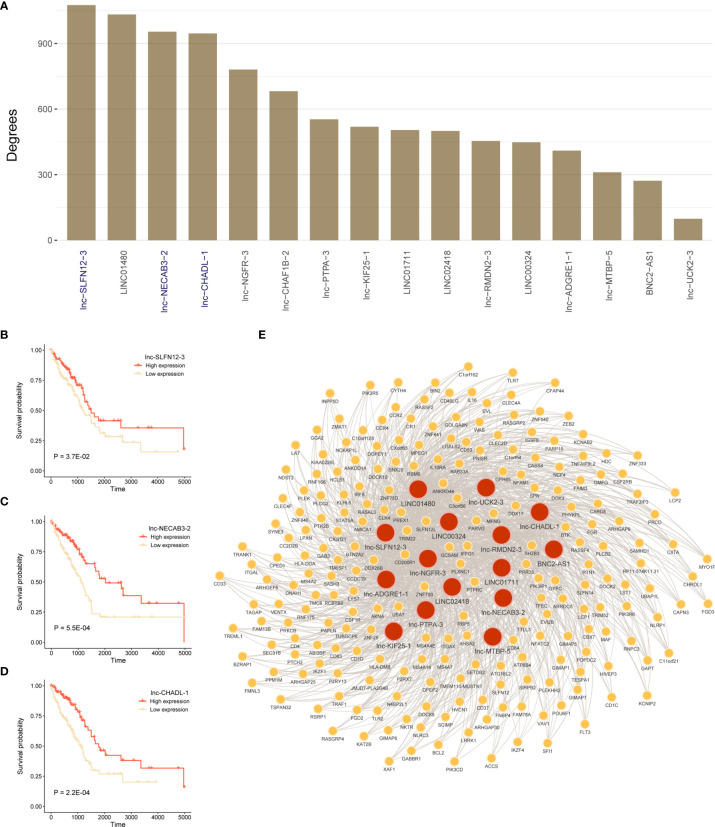
Construct lncRNA-mRNA co-expression network and survival analysis for hub lncRNAs. **(A)** Degrees of 16-lncRNAs in lncRNA-mRNA co-expression network. **(B–D)** Log-rank test was used to assess the difference in OS between lnc-SLFN12-3, lnc-NECAB3-2, or lnc-CHADL-1 high expression and low expression samples in TCGA LUAD training cohort. **(E)** Significant co-expression network between 16-lncRNAs and 225 mRNAs (keep significant co-expression relationships more than quarter 16-lncRNAs). Red nodes mean lncRNAs, and yellow nodes mean mRNAs. The width of the edge represents the Pearson correlation.

To identify mRNAs that are associated with 16 lncRNAs in prognostic model, we constructed lncRNAs and mRNAs co-expression network. **Figure 7A** showed degrees of 16-lncRNAs in the co-expression network. We found that three lncRNAs were related to prognosis in top 5 degrees lncRNAs in TCGA LUAD cohort. Patients with high expression of lnc-SLFN12-3, lnc-NECAB3-2, or lnc-CHADL-1 had a significantly better OS than those in the low expression (**Figures 7B–D**; P < 0.05; log-rank test). We visualized mRNA-lncRNA co-expression network in **Figure 7E** in which mRNAs has more than four co-expressed lncRNA partners.

### The Function of mRNAs in Co-Expression Network Involved in Immune Pathways

**Figure 8:**
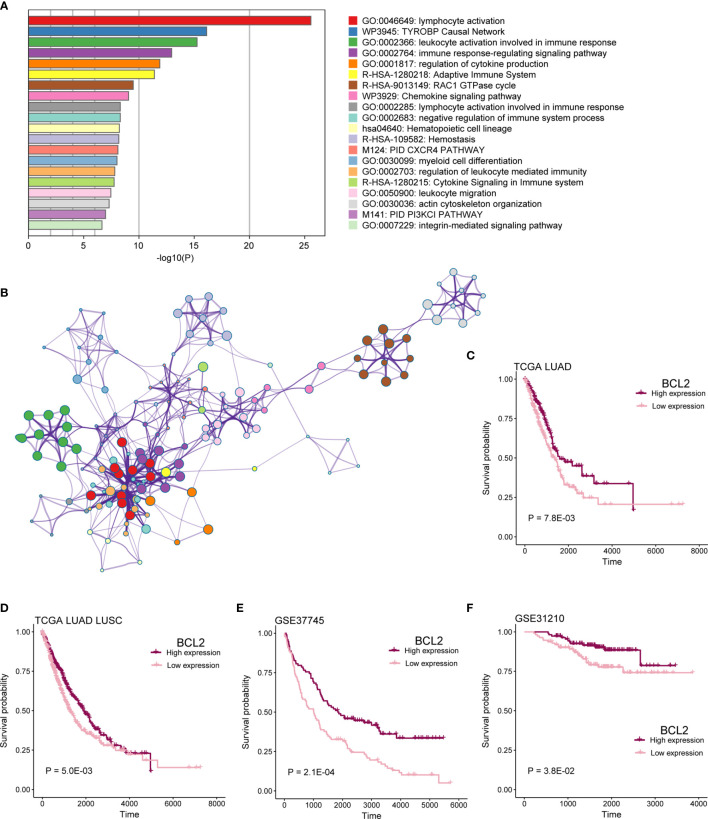
mRNAs in co-expression network functional analysis. **(A)** Enrichment analysis for 16-lncRNAs significant co-expression 225 mRNAs. The graphical representation showed top 20 enrichments with *P* < 0.01. P value was multi-test–adjusted in log base 10. **(B)** Enriched terms with a similarity > 0.3 are connected by edges. **(C–F)** Survival analysis showed the difference in OS between BCL2 high expression and low expression samples in TCGA LUAD, TCGA lung cancer, GSE37745, and GSE31210 cohorts.

To identify functional processes regulated by co-expression network comprehensively, we performed pathway and process enrichment analysis for 225 mRNAs by Metascape, including 10 pathway resources. Significant terms in biological processes were “lymphocyte activation,” “TYROBP Causal Network,” “leukocyte activation involved in immune response,” “immune response-regulating signaling pathway,” “regulation of cytokine production,” and so on (**Figure 8A**). Network plot showed the subset of enriched terms, only the terms with a similarity > 0.3 are connected by edges (**Figure 8B**).

We found that patients with mRNA BCL2 high expression had a better prognosis than low expression in TCGA LUAD, TCGA lung cancer, GSE37745, and GSE37745 cohorts (**Figures 8C–F**; P < 0.05). BCL2 is a member of the Bcl-2 protein family that regulates cell death (apoptosis) by inhibiting (anti-apoptotic) or inducing (pro-apoptotic) apoptosis (20, 21). It was the first apoptosis regulator identified in any organism.

In addition, patients with mRNA IKZF1 high expression had a better prognosis in TCGA LUAD, TCGA lung cancer, GSE37745, GSE37745, and GSE30219 cohorts (**Figure S3A–E**; P < 0.05). IKZF1 encodes the DNA-binding protein Ikaros (also known as Ikaros family zinc finger protein 1), which plays an important function in the hematopoietic system and is a regulator of early B-cell, CD4+ T-cell, and other immune cell development. It is closely associated with the development of chronic lymphocytic leukemia (22, 23).

## Discussion

For the past few years, bioinformatics analysis was widely preformed in cancer research (24). The immune microenvironment has also been shown to play an essential part in various cancers. For example, in invasive breast cancer, CD2 was an immune-related prognostic biomarker regulating the tumor microenvironment (25). MNK1/2-eIF4E regulatory axis can affect immunosuppression and metastasis in breast cancer (26). LncRNAs have also been reported to influence the prognosis of many tumors. For instance, lncRNA AGAP2-AS1 can enhance lung cancer radiotherapy immunity by regulating the expression of microRNA-296 and NOTCH2 (27). LncRNA TP53TG1 inhibits hepatocellular carcinoma growth and metastasis by affecting the PRDX4/β-catenin axis (28). The lncRNA BBOX1-AS1 is closely associated with the malignant cellular phenotype of non-small cell lung cancer. It can regulate miR-27a-5p through the ceRNA network, thereby up-regulating MELK to activate the FAK signaling pathway (29). In summary, lncRNA is non-negligible in LUAD. Nevertheless, there are few studies on immune microenvironment-related lncRNAs in LUAD.

In this study, we constructed a survival model containing 16 immune microenvironment-related lncRNAs in LUAD through ESTIMATE method and other bioinformatics analysis. This method has been shown to be feasible in cancer research and has been widely used (30, 31). A model with 16 immune- and prognosis-related lncRNAs was constructed and successfully predicted patients’ OS (**Figures 2**, **3**). Of 16 lncRNAs, *lnc-CHAF1B-2, lnc-NECAB3-2, lnc-PTPA-3, lnc-CHADL-1, lnc-RMDN2-3, lnc-SLFN12-3, lnc-UCK2-3, lnc-KIF25-1, lnc-MTBP-5, lnc-ADGRE1-1, lnc-NGFR-3* have not been reported in LUAD and other cancers. They may be novel prognostic biomarkers of LUAD. *LINC00324* have been reported to influence cell proliferation and invasion of several tumors and could regulate the IGF1R to affect non-small cell lung cancer cell invasion (32–34). LINC01711 also have prognostic ability in esophageal squamous cell carcinoma (35). *LINC01480* is a known biomarker of endometrial cancer (36). BNC2-AS1 could influence the proliferation and invasion of gastric cancer (37). LINC02418 has also been recovered to promote colon cancer progression (38).

Next, further investigation was conducted to determine whether the risk scores could indicate prognosis in different subgroups of clinical features. We found that this survival model was able to distinguish high- and low-risk groups, irrespective of gender (**Figures 4A, B**). Meanwhile, patients with early stage in TNM stage and pathological stage had higher risk scores and lower survival rates (**Figure 4**), suggesting that our survival model may have an equivalent or better efficacy in early-stage patients. In addition, we found significant differences in the expression of five immune checkpoint-related genes among the high-risk and low-risk groups of the survival model (**Figure 5**), suggesting that the model we constructed could distinguish groups that are more suitable for immunotherapy.

We then explored genomic mutation status between high- and low-risk groups. **Figures 6A, B** showed the top 20 mutations in TCGA cohort. TP53 is the most frequent mutation gene in both groups. We next investigated relationship between the mutation of TP53 and OS in high- and low-risk group and found that the mutation of TP53 indicated poor prognosis in high-risk group. TP53 mutation significantly increased the expression of immune checkpoints and activated T-effector (39–41). In this study, we also explored mutation status between high- and low-risk groups and found that TP53 was the gene with the highest mutation frequency in both risk groups (**Figures 6A, B** **)**. Then, we further explored the influence of TP53 mutation on patients’ survival. We found that patients with TP53 mutations had poor survival rates in the high-risk group, suggesting that the survival model also associated with TP53 mutation. Some studies indicated that TP53 mutation significantly increased the expression of immune checkpoints and activated T-effector and associated with poor survival (39–41). Meanwhile, we found lower immune infiltration in the high-risk group (**Figure 5**). These results indicated that patients with TP53 mutations in the high-risk group might have the potential of better immunotherapy efficacy.

Finally, our model-based co-expression network also enriched many immune-related pathways, such as immune response-regulating signaling pathway and cytokine signaling in immune system. It also suggests that lncRNAs in this model are closely related to immunity. These results showed that the biomarkers we obtained have a large research potential in LUAD.

In conclusion, we hope that the results of this study will help identify immune-related potential prognostic lncRNAs and thus provide new molecular biomarkers for improving the poor prognosis of LUAD.

## Data Availability Statement

The datasets presented in this study can be found in online repositories. The names of the repository/repositories and accession number(s) can be found in the article/**Supplementary Material**.

## Author Contributions

YM contributed to the conception and design of the review. LY performed the research and wrote the manuscript. FeL and SW revised the manuscript. All authors contributed to the article and approved the submitted version.

## Funding

This study was supported by Wu Jie Ping Medical Foundation (320.6750.2020-19-31).

## Conflict of Interest

The authors declare that the research was conducted in the absence of any commercial or financial relationships that could be construed as a potential conflict of interest.

## Publisher’s Note

All claims expressed in this article are solely those of the authors and do not necessarily represent those of their affiliated organizations, or those of the publisher, the editors and the reviewers. Any product that may be evaluated in this article, or claim that may be made by its manufacturer, is not guaranteed or endorsed by the publisher.

## Supplementary Material

Supplementary Figure 1 Workflow of this study.

Supplementary Figure 2 Distribution of survival time and risk scores in TCGA cohort. Distribution of survival time and risk scores which was calculated based on the expression of 16-lncRNA prognostic model in **(A)** TCGA LUAD and **(B)** TCGA lung cancer cohorts.

Supplementary Figure 3 Survival analysis for IKZFI. **(A–E)** The difference in OS between IKZFI high expression and low expression samples in TCGA LUAD, TCGA lung cancer, GSE37745, GSE31210, and GSE30219 cohorts.
