# Supplementary figures and images for: Identification of Tumor Microenvironment-Related Prognostic lncRNAs in Lung Adenocarcinoma
Source: Front Oncol. 2021 Aug 2;11:719812. doi: 10.3389/fonc.2021.719812 (PMC8366027; doi:10.3389/fonc.2021.719812)

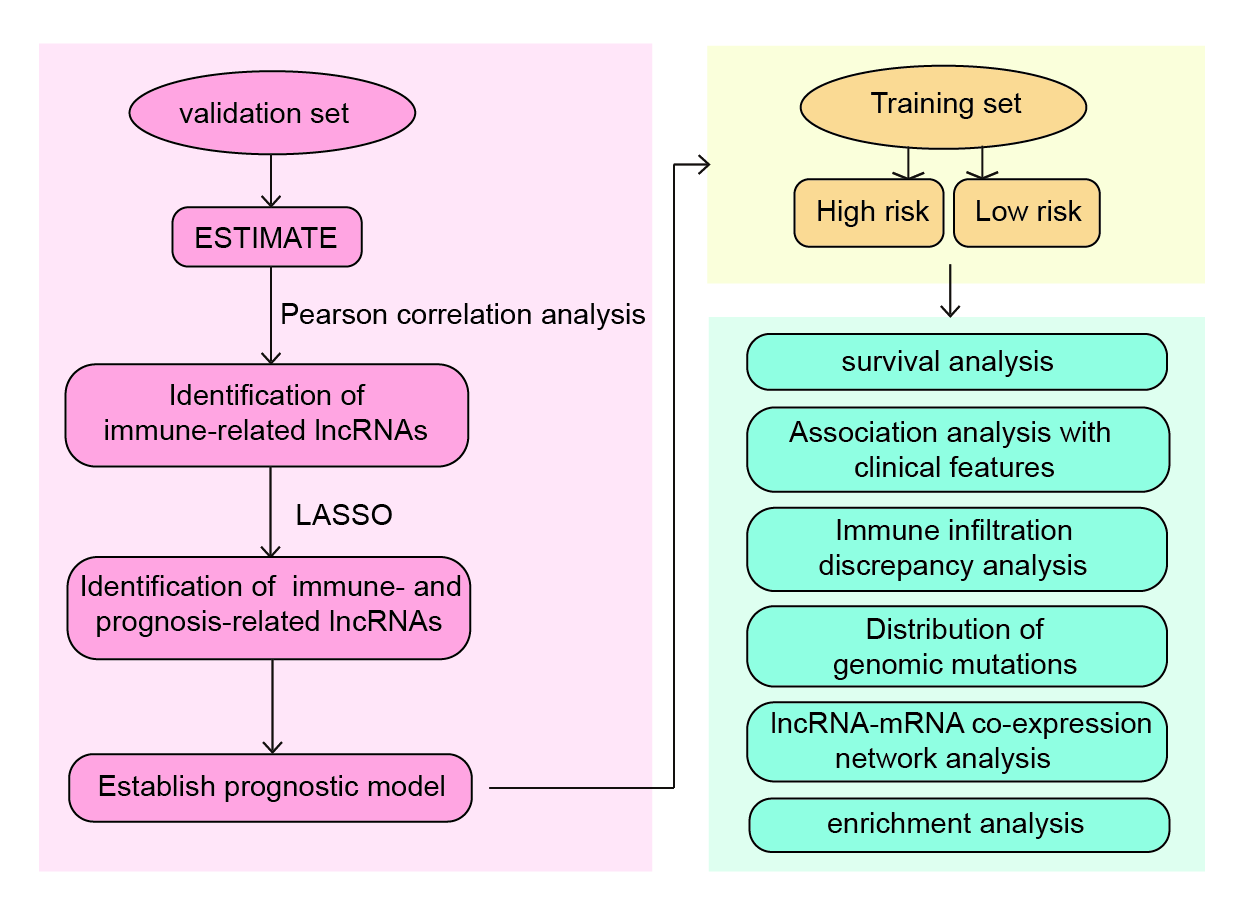

Supplement: Supplementary Figure 1 — Workflow of this study. [file Image_1.tif]

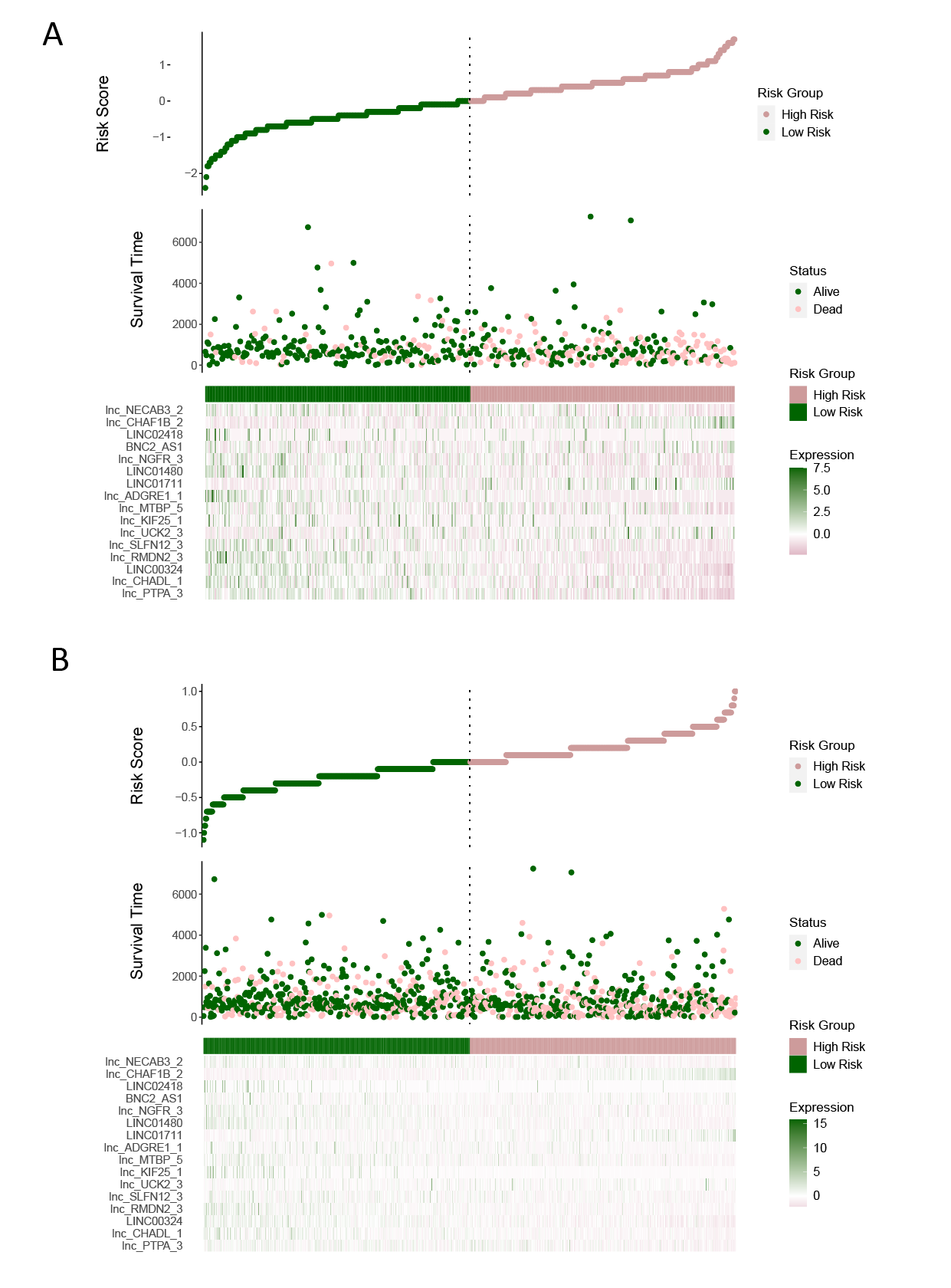

Supplement: Supplementary Figure 2 — Distribution of survival time and risk scores in TCGA cohort. Distribution of survival time and risk scores which was calculated based on the expression of 16-lncRNA prognostic model in (A) TCGA LUAD and (B) TCGA lung cancer cohorts. [file Image_2.tif]

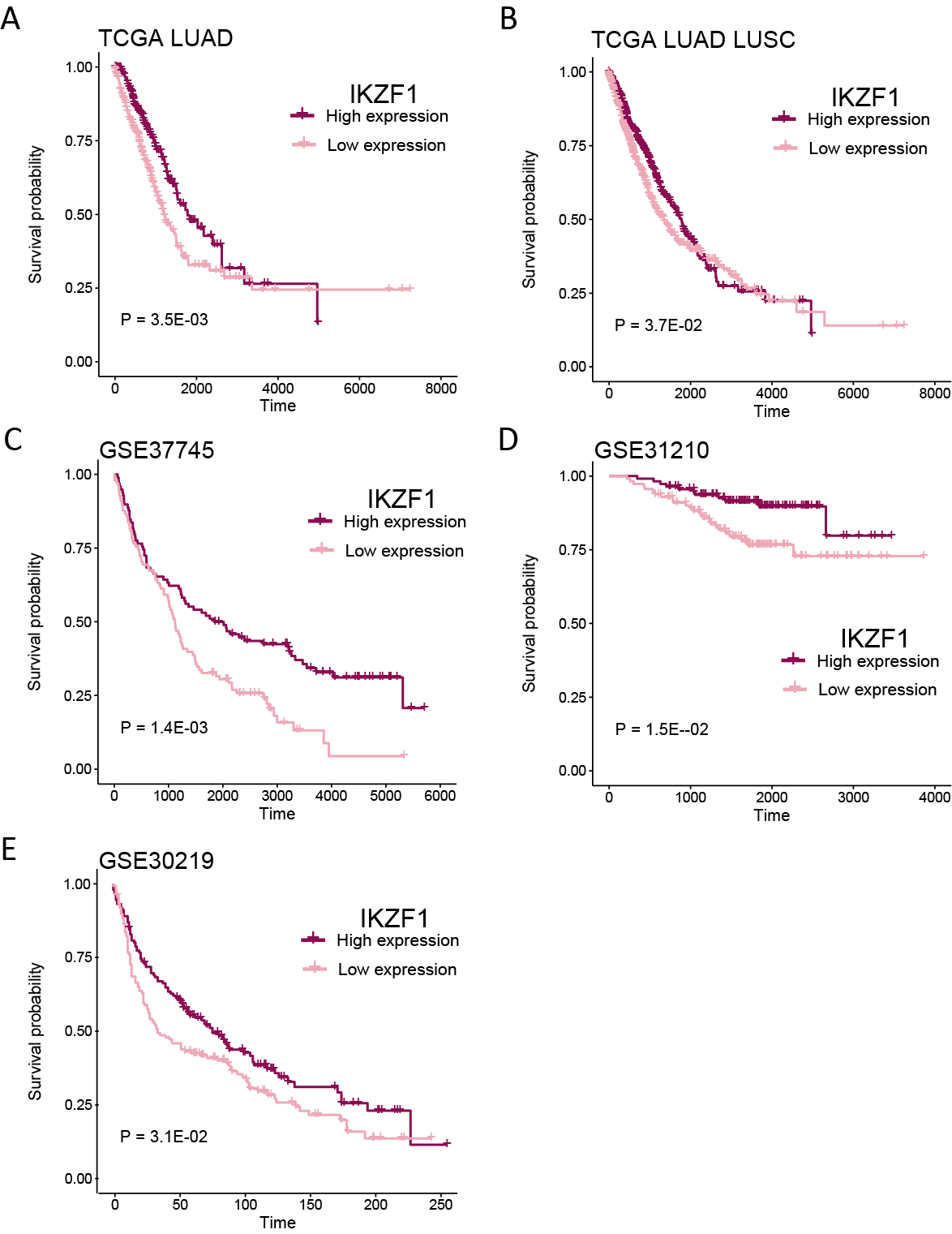

Supplement: Supplementary Figure 3 — Survival analysis for IKZFI. (A–E) The difference in OS between IKZFI high expression and low expression samples in TCGA LUAD, TCGA lung cancer, GSE37745, GSE31210, and GSE30219 cohorts. [file Image_3.tif]
