# Supplementary material for: Identification of Tumor Microenvironment-Related Prognostic lncRNAs in Lung Adenocarcinoma
Source: Front Oncol. 2021 Aug 2;11:719812. doi: 10.3389/fonc.2021.719812 (PMC8366027; doi:10.3389/fonc.2021.719812)
Supplement: Supplementary file 4 [file Table_1.docx]

**Table 1. Baseline characteristics of patients in TCGA LUAD cohort**

| **Characteristics** | **Whole cohort** | **Training set** | **Validation set** | ***p*** |
| --- | --- | --- | --- | --- |
| TCGA cohort | (n=510) | (n=255) | (n=255) |  |
| Gender |  |  |  | 0.25 |
| Male | 235(46.08%) | 124(48.63%) | 111(43.53%) |  |
| Female | 275(53.92%) | 131(51.37%) | 144(56.47%) |  |
| Age |  |  |  | 0.96 |
| <65 years | 219(42.94%) | 109(42.75%) | 110(43.14%) |  |
| >=65 years | 272(53.33%) | 136(53.33%) | 136(53.33%) |  |
| T-stage |  |  |  | 0.74 |
| T1 | 167(32.74%) | 83(32.55%) | 84(32.94%) |  |
| T2 | 276(54.12%) | 134(52.55%) | 142(55.69%) |  |
| T3 | 45(8.82%) | 25(9.80%) | 20(7.84%) |  |
| T4 | 19(3.73%) | 11(4.31%) | 8(3.14%) |  |
| N-stage |  |  |  | 0.48 |
| N0 | 327(64.12%) | 162(63.53%) | 165(64.71%) |  |
| N1 | 95(18.63%) | 50(19.61%) | 45(17.65%) |  |
| N2 | 74(14.51%) | 39(15.29%) | 35(13.73%) |  |
| N3 | 2(0.39%) | 0(0.00%) | 2(0.78%) |  |
| M-stage |  |  |  | 0.08 |
| M0 | 327(64.12%) | 172(67.45%) | 171(67.06%) |  |
| M1 | 95(18.63%) | 17(6.67%) | 8(3.14%) |  |
| Stage |  |  |  | 0.07 |
| I | 273(53.53%) | 126(49.41%) | 147(57.65%) |  |
| II | 119(23.33%) | 66(25.88%) | 53(20.78%) |  |
| III | 84(16.47%) | 41(16.08%) | 43(16.86%) |  |
| IV | 26(5.10%) | 18(7.06%) | 8(3.14%) |  |
